# The evolution of cooperative turn-taking in animal conflict

**DOI:** 10.1186/1471-2148-11-323

**Published:** 2011-11-03

**Authors:** Mathias Franz, Daniel van der Post, Oliver Schülke, Julia Ostner

**Affiliations:** 1 Courant Research Centre Evolution of Social Behaviour, University of Göttingen, Kellnerweg 6, 37077 Göttingen, Germany; 2 Institute of Artificial Intelligence, University of Groningen, PO Box 407, 9700 AK, Groningen, The Netherlands; 3 Behavioural Ecology and Self-Organization, University of Groningen, PO Box 11103, 9700 CC Groningen, The Netherlands

## Abstract

**Background:**

A fundamental assumption in animal socio-ecology is that animals compete over limited resources. This view has been challenged by the finding that individuals might cooperatively partition resources by "taking turns". Turn-taking occurs when two individuals coordinate their agonistic behaviour in a way that leads to an alternating pattern in who obtains a resource without engaging in costly fights. Cooperative turn-taking has been largely ignored in models of animal conflict and socio-ecological models that explain the evolution of social behaviours based only on contest and scramble competition. Currently it is unclear whether turn-taking should be included in socio-ecological models because the evolution of turn-taking is not well understood. In particular, it is unknown whether turn-taking can evolve when fighting costs and assessment of fighting abilities are not fixed but emerge from evolved within-fight behaviour. We address this problem with an evolutionary agent-based model.

**Results:**

We found that turn-taking evolves for small resource values, alongside a contest strategy that leads to stable dominance relationships. Turn-taking leads to egalitarian societies with unclear dominance relationships and non-linear dominance hierarchies. Evolutionary stability of turn-taking emerged despite strength differences among individuals and the possibility to evolve within-fight behaviour that allows good assessment of fighting abilities. Evolutionary stability emerged from frequency-dependent effects on fitness, which are modulated by feedbacks between the evolution of within-fight behaviour and the evolution of higher-level conflict strategies.

**Conclusions:**

Our results reveal the impact of feedbacks between the evolution of within-fight behaviour and the evolution of higher-level conflict strategies, such as turn-taking. Similar feedbacks might be important for the evolution of other conflict strategies such as winner-loser effects or coalitions. However, we are not aware of any study that investigated such feedbacks. Furthermore, our model suggests that turn-taking could be used by animals to partition low value resources, but to our knowledge this has never been tested. The existence of turn-taking might have been overlooked because it leads to societies with similar characteristics that have been expected to emerge from scramble competition. Analyses of temporal interaction patterns could be used to test whether turn-taking occurs in animals.

## 1. Background

A fundamental assumption in animal socio-ecology is that animals compete with each other over limited resources such as food or mates. Two basic forms of competition are usually distinguished: scramble and contest [1]. Scramble competition should occur when individuals are not able to prevent others from accessing a resource. In contrast, contest competition occurs when individuals can directly influence others' access to a resource, e.g. by winning fights over monopolizable resources. The form of competition should affect how individuals interact with each other, which in turn should determine the development of social relationships and social structures in group living animals [2]. This is a central assumption in conceptual socio-ecological models that have been developed for primates [3-6]. In this framework, a high potential for contest competition within groups leads to despotic societies with stable, unidirectional dominance relationships and linear hierarchies. In contrast, when the potential for contest competition is low and individuals mainly compete via scramble competition, egalitarian societies should emerge with unstable dominance relationships and non-linear hierarchies.

The assumption that animals always use competitive mechanisms to gain access to limited resources has been challenged by Crowley [7], who discovered a potential cooperative mechanism for this purpose. Crowley's study was based on a generalized hawk-dove game in which individuals of different size repeatedly enter conflicts with each other over a valuable resource. In each conflict two individuals decide whether to play hawk (i.e., to escalate to a costly fight) or dove (i.e. not to escalate), which determines whether the conflict is escalated to a costly fight. In particular, Crowley considered conflict strategies that were conditional on interactions in previous conflicts with the same opponent. He found that two conditional strategies evolved: a "contest strategy" and a "turn-taking strategy". The contest strategy resulted in stable dominance relationships with fixed hawk and dove roles. The turn-taking strategy resulted in alternating dominance interactions. In each conflict, always one individual in a dyad played hawk and the other played dove. Importantly, in each subsequent conflict between the same individuals both individuals switched their roles of playing hawk and dove. Thus, in the long term, turn-taking resulted in the equal partitioning of resources despite the fact that contest competition was possible. According to Noë's [8] definition of cooperation, turn-taking is a cooperative strategy because it leads to net gain for both individuals in each dyad.

While the evolution of turn-taking has been investigated in cooperation games [9,10], turn-taking has been usually ignored in models that explored the evolution of conflict strategies [11-16] and the emergence of social structures such as dominance hierarchies [17-20] and coalitions [18,21-24]. Also, turn-taking was not included in socio-ecological models that explain the emergence of egalitarian and despotic societies based only on contest and scramble competition [4-6]. Nevertheless, turn-taking might be an important determinant of social structures. Specifically it could be a previously unrecognized mechanism for the emergence of egalitarian societies [7]. However, currently it is largely unclear whether turn-taking should be integrated in socio-ecological models because it is not well understood under which conditions turn-taking can evolve.

Crowley [7] found that the evolution of cooperative turn-taking in his model is favoured if costs of fights are not very high and if individuals are not able to assess each other's fighting abilities (or when individuals have very similar fighting abilities, see also [25]). However, these findings were based on models in which fighting costs and the assessment of the opponent's fighting abilities are fixed attributes of a species. As shown by Enquist and Leimar [26] costs and assessment are not necessarily fixed, because they can depend on evolved within-fight behaviour. In their "sequential assessment game" Enquist and Leimar [26] assumed that in the course of a fight, individuals assess each other's strength and give up when they are certain to be weaker. While longer persistence in fights allows a better assessment of fighting abilities, it also increases fighting costs. Based on these assumptions Enquist and Leimar [26] found that the evolution of within-fight behaviour depended (among other factors) on the value of the contested resource. Accordingly, lower resource values resulted in shorter fights that are less costly and allow an inferior assessment of fighting abilities. Given these findings we might expect that turn-taking evolves when the value of the limited resource is small.

However, this expectation could be wrong because in the model of Enquist and Leimar [26] within-fight behaviour evolves independently of any higher-level strategy, such as the turn-taking, or the contest strategy found by Crowley [7]. Enquist and Leimar assumed that individuals do not adjust their behaviour based on past experiences. In contrast, if within-fight strategies evolve together with higher-level strategies, feedbacks between the two evolutionary processes might change the dynamics that are expected when both processes occur in isolation. To our knowledge such feedback effects have never been investigated in models of animal conflict. Therefore, it is unknown whether such feedback effects exist and what consequences they could have. In this study we address this problem with an evolutionary agent-based model. For this purpose we formulate a model which is a hybrid of Crowley's [7] and Enquist and Leimar's [26] models. Thus, we assumed that individuals can recognize each other individually and are able to react to experiences in conflicts by adjusting future dyad-specific conflict behaviour. In addition, we assumed that costs of fights and assessment of fighting abilities are not fixed, but that they emerge from evolving within-fight behaviour. In our model analysis we were particularly interested in whether turn-taking can be evolutionarily stable when evolving within-fight behaviour determines fighting costs and assessment of fighting abilities.

## 2. Methods

### 2.1. Model description

The description of our model is based on the ODD protocol for describing individual- and agent-based models [27,28]. In the following we provide an overview. Information about details is included in Appendix A.

#### 2.1.1. Purpose

The main purpose of this model was to explore the evolution of conflict strategies at two different levels: (1) how to behave in escalated conflicts (i.e. fighting behaviour in costly fights) and (2) how to adjust conflict behaviour in a series of conflicts (i.e. higher-level conflict strategies). Specifically, we wanted to investigate whether and under which conditions cooperative turn-taking (which occurs at the second level) can be evolutionarily stable.

#### 2.1.2. State variables and scales

We assume that individuals live in stable groups of equal size. Each individual is characterized by its strength *s *(which differs among individuals and influences fighting costs) and fitness *F*. In addition, each individual *i *has a set of dyad-specific variables *w*_*i,j *_that quantify the willingness of *i *to escalate a conflict with group member *j *into a costly fight. Individuals can adjust their dyad-specific willingness to escalate based on the outcomes of conflicts. We assume that a genetically determined update factor *U *regulates how winning or losing changes the dyad-specific willingness to escalate. In the case that costly fights occur, individuals make use of two additional variables: a decision variable *d*, which contains information about their estimated relative strength, and a genetically determined threshold *T*, which determines when to give up.

#### 2.1.3. Process overview and scheduling

**Figure 1:**
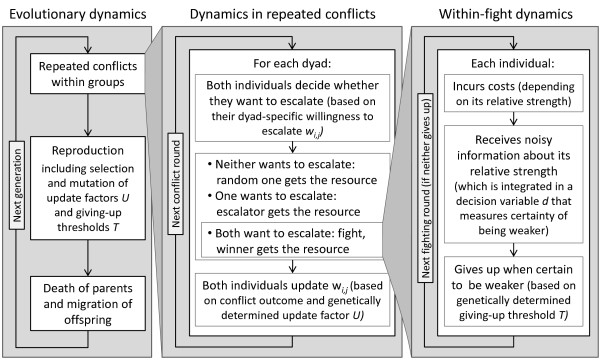
**Overview of model dynamics that proceed on three different timescales: evolutionary dynamics, (2) dynamics in repeated conflicts and (3) within-fight dynamics, which are nested in each other**.

Model dynamics include dynamics at three different time scales: (1) evolutionary dynamics that occur over several generations, (2) dynamics of repeated conflicts among individuals of one generation and (3) if a conflict is escalated into a costly fight then also within-fight dynamics occur (figure 1). Evolutionary dynamics proceed in discrete generations, in which three distinct phases occur: repeated conflicts among group members (which determine the fitness of individuals), reproduction (which includes fitness-dependent selection and mutation) and death of parents and migration of offspring.

Dynamics of repeated conflicts proceed in distinct rounds in which all dyads in a group enter a conflict over a valuable resource. In each conflict only one individual can obtain a resource and the associated fitness benefit. When a conflict is escalated into a costly fight then both individuals also incur fitness costs. Each conflict starts with both individuals simultaneously deciding whether they are willing to escalate the conflict into a costly fight. The probability with which an individual *i *makes this decision regarding an individual *j *is given by the value of the dyad-specific willingness to escalate *w*_*i,j*_,. We assume that individuals make use of specific signals to communicate their willingness to escalate the conflict (although this is not an essential assumption). If neither individual is willing to escalate, then one randomly chosen individual obtains the resource. If only one individual is willing to escalate, then this individual obtains the resource. In case both are ready to escalate, a fight occurs in which both individuals suffer costs and only the winner obtains the benefits. Based on conflict outcomes, individuals adjust their future behaviour by updating their willingness to escalate in the next conflict *w*_*i,j *_(based on their update factor *U*).

Within-fight dynamics take place in discrete fighting rounds in which individuals obtain noisy information about their relative strength (the strength of noise is determined by the values of its standard deviation σ_ε_) and incur costs. The obtained information about relative strength is integrated into the decision variable *d *that measures the certainty of being weaker (which is measured independently of previous fights, i.e. starts out at zero). An individual gives up when sufficiently certain to be weaker (i.e. when the value of *d *increases above the giving-up threshold *T*). The costs of a fight depend on the relative strength of individuals (costs are higher for the weaker individual) and the length of the fight (which strongly depends on the genetically determined giving-up threshold *T*). More details about the model are included in Appendix A.

### 2.2. Model analysis

In the main analysis we focussed on a specific model parameterization and explored model dynamics in more detail in additional analyses that are provided in Appendix B. In the main analysis the number of groups was set to 1000 with 10 individuals per group, 10 conflict rounds and an initial fitness of individuals of 100. The standard deviation σ_ε _of errors in estimating the relative strength in each fighting round was set to one. Simulations were performed while systematically varying values for resource benefits (from 1 to 10 in steps of 1) and initial values of the update factor *U *(which was either set to -1 or 1). The initial value for the giving up threshold *T *was set to zero in all simulations. Simulations for each parameter combination were repeated ten times (resulting in a total of 200 simulations). Each simulation was run for 5000 generations. In the last generation of each simulation we recorded the values of *U *and *T *of all individuals. In addition, we recorded the length of all fights and calculated the directional consistency index (DCI), which measures directionality of a dyadic relationship [29], and linearity of the hierarchy in each group [30]. The calculation of DCI and linearity was based on outcomes of conflicts in which at least one individual was ready to escalate.

Our results revealed that two alternative strategies evolved for small benefit values. To investigate whether both strategies were evolutionarily stable, we performed competition experiments to determine whether each strategy could resist the invasion of the alternative strategy. Two sets of experiments were performed in which individuals were initialized with the means of evolved parameters *U *and *T *for the two strategies. The first set of experiments was based on parameters that evolved for benefit values of one and the second set was based on parameters that evolved for benefit values of four. In both sets we performed simulations with group sizes of two (with 100,000 groups), to show the effects of direct competition in dyads, and group sizes of ten (with 20,000 groups), to reveal effects of indirect competition among dyads. Different simulations were performed for different frequencies of both strategies. For this purpose, in each group we initialized the same number of individuals with each strategy. Benefits were set to one and four in the first and second set, respectively. For each group size and strategy frequency one simulation was run for one generation. At the end of each simulation we recorded the mean fitness of individuals for each strategy.

## 3. Results

### 3.1 Evolutionary dynamics

**Figure 2:**
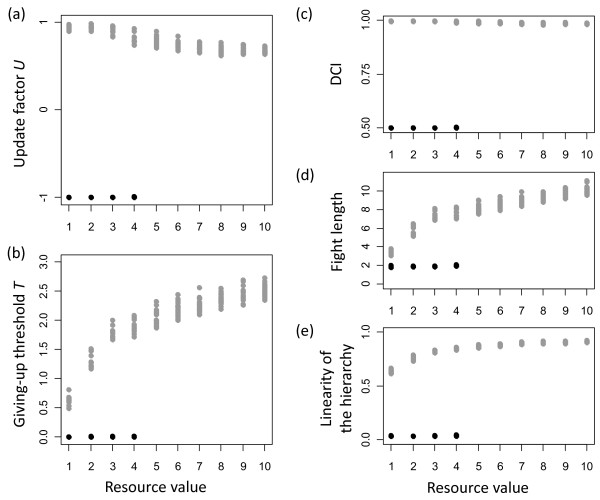
**Evolved parameters (a, b) and resulting behavioural dynamics (c, d, e)**. For small resource values two distinct evolutionary outcomes occur, which correspond to turn-taking (black dots) and contest strategies (grey dots). The evolution of a positive update factor *U *(a) results in the establishment of stable subordinate-dominant relationships, which are characterized by high values of the directional consistency index (DCI) (c). In contrast, the evolution of a negative update factor *U *leads to the establishment of alternating dominant-subordinate relationships, which are characterized by low DCI values (c). Higher evolved values of the giving-up threshold *T *(b) result in longer fights (d). This increases the likelihood that stronger individuals win (figure 4 in Appendix A), which leads to more linear hierarchies (e).

Simulated evolutionary dynamics differed between small and large values of resource benefits. While for large benefits, all simulations led to similar evolutionary outcomes, for small benefits two distinct evolutionary outcomes emerged (figure 2 a, b). Based on emerging behavioural dynamics we refer to these distinct outcomes as contest and turn-taking strategies.

The contest strategy is characterized by positive values of the update factor *U *(figure 2 a) and high values of the giving-up threshold *T *(figure 2 b). At positive values of *U *winning a conflict leads to an increase in willingness to escalate the next conflict with the loser and losing leads to a decrease in willingness to escalate the next conflict with the winner (for more information on the effects of *U *see Appendix A). Based on this effect, stable dominance relationships emerge in which only the dominant individual is willing to escalate in conflicts and thus always obtain the resource. This behavioural pattern leads to very high values of the directional consistency index (DCI) (figure 2 c). The high giving-up threshold of the contest strategy has the effect that individuals collect substantial evidence for being weaker before giving up in an escalated fight. Therefore, the contest strategy leads to relatively long (and costly) fights (figure 2 d). Higher giving-up thresholds also increase the likelihood for stronger individuals to win fights and thus to become dominant. Therefore, the high giving-up threshold of the contest strategy result in the establishment of linear hierarchies (figure 2 e). With increasing resource benefits the giving-up threshold *T *of the contest strategy increase, which results in increased fight lengths and increased linearity of the hierarchy. The update factor *U *decreases with increasing benefits, which has the effect that dyads do not resolve dominance in a single fight. Instead, several fights with consistent outcomes are required to establish stable relationships. Therefore, even individuals that are only slightly stronger than their opponent have a high likelihood of becoming dominant, which contributes to increasing hierarchy linearity with increasing benefits.

The turn-taking strategy is characterized by strongly negative values of the update factor *U *(figure 2 a) and low values of the giving-up threshold *T *(figure 2 b). At negative values of *U *winning a conflict leads to a decrease in willingness to escalate in the next conflict with the loser, and losing leads to an increase in willingness to escalate the next conflict with the winner. In the turn-taking strategy this effect is so strong, that interaction patterns emerge in which in each conflict one individual behaves dominantly (because it is willing to escalate) and the other behaves subordinately (because it is not willing to escalate). Importantly, these roles switch in each subsequent conflict because the "dominant" individual won and thus decreases its dyad-specific willingness to escalate to zero (or very close to zero) and the "subordinate" individual increases its willingness to one (or very close to one). As a result of this emerging alternating pattern, DCI values are very low (figure 2 c) and no linear hierarchies emerge (figure 2 e). Because the values of the giving-up threshold are very low, individuals give up very quickly and fights are short (figure 2 d).

### 3.2. Competition experiments

**Figure 3:**
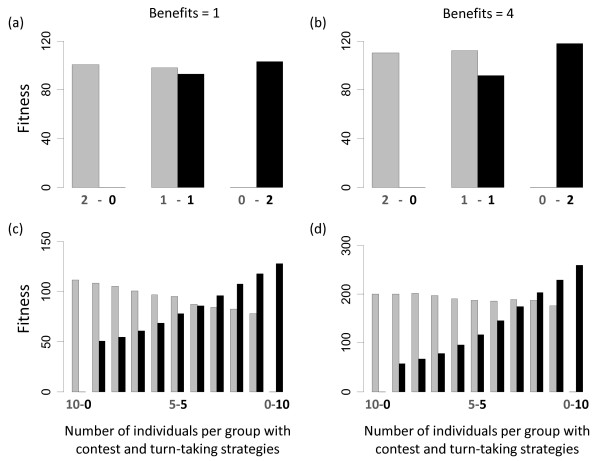
**Results of competition experiments**. Mean fitness of contest (grey) and turn-taking (black) strategies that evolved for benefit values of one (a, c) and four (b,d). (a,b) in groups of size two (which illustrates effects of direct competition in dyads) and ten (which are conditions under which these strategies evolved). For each group all possible frequencies of the two strategies were investigated (the number of individuals with the turn-taking strategy increases from the left to the right).

Results of the competition experiments reveal why the two different strategies are evolutionarily stable and why the turn-taking strategy only evolves for small benefit values. When the two strategies compete with each other in dyads, the contest strategy obtains on average a higher fitness than the turn-taking strategy (figure 3 a, b). The costs and benefits for individuals of each strategy strongly depend on the strength differences between competing individuals. Nevertheless, because individuals with the contest strategy are more persistent in fights (due to a higher giving-up threshold), they are able to obtain the resource on average more often than turn-taking individuals. Therefore, in direct competition with the contest strategy the turn-taking strategy had a disadvantage. However, when turn-taking individuals compete with each other they achieve a higher average fitness than individuals with the contest strategy (figure 3 a, b). This happens because individuals with the turn-taking strategy rarely engage in fights with each other (usually only when they entered a conflict for the first time) and if they did fights are very short (figure 2 d) and not very costly. Together, this leads to frequency-dependent effects on the fitness of alternative strategies (figure 3 c, d). If the number of turn-taking individuals in a group is low then contest individuals achieve on average a higher fitness than turn-taking individuals, because turn-taking individuals mostly encounter contest individuals. The opposite effect emerges when the number of turn-taking individuals is high. The fitness advantages that turn-taking individuals gain in conflicts with individuals of their own strategy outweigh the disadvantages they incur in conflicts with contest individuals. Due to the frequency-dependent fitness, evolutionary dynamics result in a positive feedback on the frequency of each strategy and prevent the invasion of the alternative strategy. In other words, the social behaviour of each strategy is able to create a social environment that favours its own strategy and thus makes both strategies evolutionarily stable.

With increasing benefit values, the contest strategy becomes more successful because individuals with this strategy achieve increasing fitness advantages in direct competition with turn-taking individuals (compare figure 3 a, b). Therefore, for benefit values of four the turn-taking strategy is only more successful if the frequencies of turn-taking individuals are very high (compare figure 3 c, d). This effect can be also described as a decreasing potential of the turn-taking strategy to create a social environment that favours its own strategy, which explains why the turn-taking strategy does not evolve for benefit values larger than four (figure 2).

### 3.3. Additional analyses

We found that the results reported above remain qualitatively unchanged when changing the number of individuals in groups, the number of conflicts and the standard deviation of errors in estimating relative strength (Appendix B for more details). In addition, the range of benefit values under which the turn-taking strategy evolves increase with increasing observation errors, a decreasing number of contest rounds and an increasing number of individuals per group. These effects emerge because variations in the investigated parameters change the effectiveness with which the stronger individual in each dyad can be identified. Generally, a decreasing ability to identify the stronger individual favours the evolutionary stability of the turn-taking strategy.

## 4. Discussion

Using an evolutionary agent-based model we investigated the evolution of conflict behaviour defined at two different levels: (1) how to behave during escalated conflicts (i.e. costly fights) and (2) how to adjust conflict behaviour in a series of conflicts. This approach extends previous approaches to model the evolution of conflict behaviour that focus on only one of these levels.

In agreement with findings of Crowley [7], we found that two distinct behavioural strategies evolve: a contest strategy and a turn-taking strategy. While the contest strategy leads to stable dominance relationships, the turn-taking strategy leads to dyadic interaction patterns in which individuals constantly switch roles of who is dominant and subordinate. Thus, both strategies function to coordinate interactions to avoid costly fights but they do so in different ways. Also in agreement with findings of Enquist and Leimar [26], we observed that for the contest strategy increasing benefits lead to more persistent within-fight behaviour, as indicated by higher values of the giving-up threshold T (figure 2 b). However, for the turn-taking strategy we did not find this pattern. Instead, we found that turn-taking is always associated with very short persistence in escalated fights, independently of resource benefits (figure 2 b).

**Figure 4:**
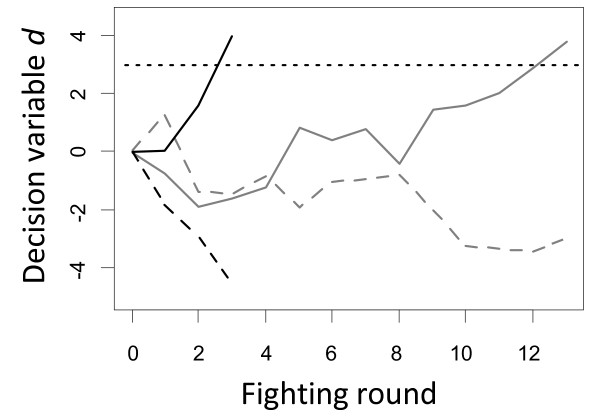
**Examples of decision making processes during fights**. Black and grey lines depict two separate, independent fights. In the first fight (black data) the strength difference between both individuals (*s*_*i *_*- s*_*j*_) was set to 1, and in the second fight (grey data) it was set to 0.3. In both fights the stronger individual is represented by a dashed line and the weaker one by a solid line. In each fighting round both individuals obtain a noisy estimation of their relative strength. This estimation is subtracted in each round from the decision variable *d *(on the y-axis), which measures the evidence of being weaker. Therefore, the decision variable of stronger individuals (dashed lines) tends to decrease over time and for weaker individuals it (solid lines) tends to increase. An individual is certain to be weaker and gives up when the value of *d *increases above the genetically determined giving-up threshold *T*, which in these examples is assumed to be three for all individuals (dotted line). The increase or decrease in the decision variable *d *is not always consistent because of the noise in the estimation of relative strength. In addition, the development of *d *strongly depends on the strength difference between both individuals. Thus, on average a smaller strength difference leads to longer fights as indicated by the differences in fight between black (3 rounds) and grey data (13 rounds). Also note that a higher giving-up threshold leads on average to longer fights, but also to a lower risk that the stronger individual erroneously becomes certain to be weaker and gives up.

The difference in within-fight behaviour between contest and turn-taking strategies emerges because escalated fights evolve to have different purposes in both strategies. Contest individuals use fights to identify opponent strength. The stronger individual in each dyad then becomes dominant, which enables coordinated interactions that avoid costly fights. Higher giving-up thresholds reduce the chance that the stronger individual erroneously assumes itself to be weaker and gives up (figure 4 in Appendix A). Therefore, contest individuals benefit from higher giving-up thresholds. However, higher thresholds also lead to longer, more costly fights. The evolution of within-fight behaviour in contest individuals evolves according to a trade-off between opponent assessment and fighting costs. With increasing benefit values, individuals can invest in more costly within-fight behaviour, which explains why for the contest strategy the giving-up threshold increases with increasing resource benefits.

Turn-taking individuals also initially fight each other. However, these fights are not used to assess opponent strength. Instead they trigger a pattern of behaviour alternation that ensures an equal partitioning of resources. Because accurate assessment of fighting abilities is irrelevant for turn-taking individuals, they do not face the same trade-off as contest individuals. This explains why the turn-taking strategy is associated with very low giving-up thresholds, independently from resource benefits.

For low resource values both strategies are possible, but mutually exclusive, evolutionary outcomes. The above described feedback between the evolution of higher-level conflict strategies and within-fight behaviour is a crucial determinant for this outcome. At low benefit values, evolutionary stability of each strategy emerges because of frequency-dependent effects (figure 3). Both strategies are very efficient in same-strategy interactions because after initial fights conflicts are resolved by ritualized interactions (in which only one of the two individuals signals willingness to escalate). In contrast, interactions between the two alternative strategies often result in on-going costly fights because turn-taking, but not contest individuals, constantly try to switch roles of dominant and subordinate (resulting in costly fights for both individuals).

With increasing benefit values this frequency-dependent effect falls more in favour of the contest strategy, because the contest strategy becomes increasingly successful in direct interactions with the turn-taking strategy (figure 3 a, b). Increasing benefits lead to increasing difference in giving-up thresholds between the turn-taking strategy and the contest strategy (figure 2 b). Large differences in giving-up thresholds increase the chance that contest individuals win fights, because contest individuals only give-up when being very certain to be weaker. However, turn-taking individuals give up much more quickly. Therefore, the contest strategy becomes increasingly successful in direct interactions with turn-taking individuals (figure 3 a, b). At large benefits the success of contest individuals increases so strongly that the frequency-dependent effects that stabilize turn-taking completely disappear and turn-taking is no longer evolutionarily stable.

Taken together, our results emphasize that the evolution of within-fight behaviour and the evolution of higher-level conflict strategies can crucially influence each other. Specifically, turn-taking is only stable for small-benefit values because with increasing benefits, the contest strategy, but not the turn-taking strategy, evolves more persistent within-fight behaviour, which makes the contest strategy more successful. This feedback between within-fight behaviour and higher-level conflict strategies could also influence other conflict strategies such as winner-loser effects (which are not restricted to dyadic interactions as the strategies in our model) [14,20,31], eavesdropping [12] or coalition formation [18,21-24]. However, only few models have been used to explore the evolution of within-fight behaviour [26,32] and we are not aware of any study that explored feedbacks between the evolution of within-fight behaviour and higher-level conflict strategies.

Furthermore, more theoretical and empirical studies would be helpful to assess our model assumptions and to develop improved models. Important points to address include assumptions about within-fight dynamics (e.g. how individuals decide to give up), which information gained in fights is used in higher-level strategies (e.g. only whether an individual lost or won, or also how long a fight lasted) and how these strategies in turn affect within-fight behaviour (e.g. should only the willingness to escalate be updated or also the giving-up threshold). In addition, instead of assuming equally frequent interactions among all group members, future studies could allow that interaction patterns emerge from self-organization processes [31].

### 4.1. Implications for socio-ecological models

It has been suggested that if contest potential exceeds a certain threshold then individuals will always rely on contest competition and form despotic societies with clear dominance relationships and linear hierarchies [5]. Our results contradict the assumption that a clear threshold exists for the emergence of despotic societies. We found that the turn-taking and the contest strategy are both evolutionarily stable for small benefits. This suggested that under these conditions despotic and egalitarian societies are both possible. To determine whether turn-taking should be included in socio-ecological theories requires investigating whether this strategy is indeed used by animals. Laboratory experiments revealed that animals and humans are able to use turn-taking strategies [33,34]. However, in the context of regulating access to limited, monolpolizable resources, we are not aware of any study that has tried to look for, nor found, any evidence of turn-taking.

The existence of turn-taking might have been overlooked because it produces similar social patterns as expected when individuals compete via scramble competition. It has been argued that individuals that compete via scramble competition would occasionally engage in context-dependent, short fights, which lead to unclear dominance relationships and non-linear hierarchies [4]. These are the same characteristics that emerge from the turn-taking strategy in our model (figure 2 c, d, e). Distinguishing turn-taking from context-dependent fights could be possible by investigating dyadic interaction patterns on small time scales. If animals compete mainly via scramble competition and only engage in context-dependent fights then we expect a rather random pattern of dominance interactions. In contrast, turn-taking should result in much more ordered patterns of these interactions. One possible way to test for such differences is to fit alternative Markov models to observed interaction sequences (e.g. [33]).

## 5. Conclusions

We found that turn-taking is evolutionarily stable for small benefit values despite strength differences among individuals, and the possibility for good assessment of fighting abilities. In our model, the lack of opponent-assessment during fights is not imposed as assumed by Crowley [7]. Instead, the lack of assessment in the turn-taking strategy is an evolved behavioural feature, which evolves even when opponent assessment is possible. This suggests that the evolution of turn-taking can be expected under a broader range of conditions than previously thought. For animals that are in principle able to assess fighting abilities of opponents, our model suggests that turn-taking could be used to partition low value resources. However, to our knowledge this has never been tested. Analyses of temporal interaction patterns could be used to test whether turn-taking occurs in animals.

Our finding of feedbacks between the evolution of within-fight behaviour and the evolution of higher-level conflict strategies contradicts the common assumption that within-fight behaviour and higher-level conflict strategies evolve independently of each other. Similar feedbacks might be important for the evolution of other conflict strategies such as winner-loser effects or coalitions. However, we are not aware of any study that has investigated such feedbacks.

## Authors' contributions

MF conceived of the study, participated in the study design, developed the model, performed model analyses and participated in drafting the manuscript. DVDP, OS and JO participated in the design of the study and in drafting the manuscript. All authors read and approved the final manuscript.

## Appendix A: Model details

### Evolutionary dynamics

Individuals are assumed to reproduce asexually, which involves two separate processes: (1) selecting individuals for reproduction based on their fitness and (2) creating offspring, which includes mutation of evolving parameters *U *(update factor) and *T *(giving-up threshold). Selection of individuals for reproduction is performed separately in each group. The number of reproduction events in each group equals the number of individuals (of the parent generation) in each group. Each time one individual of the parent generation is chosen for reproduction. Selection is assumed to be a probabilistic process that depends on the relative fitness of individuals. For each reproduction event, the probability *P*_*i *_that individual *i *is selected is given by:

(1) $$P_{i}=\frac{F_{i}}{\underset{j}{\sum }F_{j}}$$

where *F*_*i *_and *F*_*j *_are the fitness values of individuals *i *and *j*. The summation over *j *includes all individuals of the parent generation in the group. (Any negative fitness values are set to zero before selection processes are started.) Each reproduction event results in the generation of an offspring individual, which inherits the parameters *U *and *T *from the selected parent. Additionally, mutation takes place with probability 0.001. In case mutation occurs, parameters *U *and *T *are assigned new values, which are drawn from uniform distributions with a minimum of minus one and maximum of one for *U *and a minimum of zero and maximum of five for *T*. (In an earlier model version we assumed that mutations only lead to small changes in parameter values; this, however, did not affect the model outcomes). The initial fitness of an offspring individual is set to a predefined value *F*_*0*_, its strength is drawn from a uniform distribution with minimum zero and maximum one and all values of the willingness to escalate *w*_*i,j *_are set to one. After reproduction takes place all individuals of the parent generation are assumed to die. Thereafter, all individuals of the offspring generation migrate to a randomly selected group (all group sizes remain restricted to the predefined group size, which also ensures that the number of groups remains constant).

### Dynamics of repeated conflicts

Dynamics in single conflicts consist of discrete time steps in which individuals in a dyad simultaneously make decisions how to proceed. The first decision determines whether an individual is willing to escalate the conflict into a costly fight. The probability that an individual *i *is willing to escalate a conflict with another individual *j *is given by the value of *w*_*i,j*_. If neither is willing to escalate then one randomly chosen individual obtains the resource and the associated benefits (and neither suffers any costs). If only one individual decides to escalate, then this individual obtains the benefit (and again neither suffers any costs). In case that both are ready to escalate, a fight occurs in which both individuals suffer costs (according to their relative strength and the length of the fight) and only the winner obtains the benefits (below a detailed description of within-fight dynamics is provided).

We assume that each individual *i *updates its dyad-specific willingness to escalate *w*_*i,j *_based on the outcome of a conflict with each other individual *j*. Updating only occurs when at least one individual is willing to escalate. In this case, two outcomes are distinguished: (1) obtaining the resource, which can be interpreted as winning, and (2) not obtaining the resource, which can be interpreted as losing. We assume that a winner *i *updates its willingness to escalate *w*_*i,j *_for a loser *j *based on its current value *w*_*cur,i,j *_by:

(2) $$w_{i,j}=w_{cur,i,j}+U_{i}\left(1-w_{cur,i,j}\right)\;forU_{i}>0$$

and

(3) $$w_{i,j}=w_{cur,i,j}+U_{i}w_{cur,i,j}\;forU_{i}<0$$

where *U*_*i *_is the genetically determined update factor of individual *i*. The opposite effects are assumed for losers. Thus, a loser *i *adjusts its escalation probability *w*_*i,j *_for a winner *j *by:

(4) $$w_{i,j}=w_{cur,i,j}-U_{i}w_{cur,i,j}\;forU_{i}>0$$

and

(5) $$w_{i,j}=w_{cur,i,j}-U_{i}\left(1-w_{cur,i,j}\right)\;forU_{i}<0$$

In addition, we assume that when *U*_*i *_is zero, individuals will not adapt future agonistic behaviours. Our assumptions furthermore imply that positive values of *U*_*i *_will have the effect that winners increase their escalation probability and losers decrease it (which can lead to the establishment of stable dominance relationships). Negative values of *U*_*i *_have the opposite effect (which can lead to alternating roles of who appears to be dominant and subordinate).

Note that we assume that winning and losing only affects the dyad-specific willingness to escalate. More general winner-loser effects [14,20,31] are not implemented, i.e. it is not possible that winning or losing against one individual affects the willingness to escalate a conflict with another individual.

Also note that we assume that winning or losing only affects the willingness to escalate a conflict into a physical fight, but it does not affect how individuals behave within fights (i.e. the giving-up threshold *T *and the initial value of the decision variable *d *are not affected). This means that individuals do not integrate information of different fights to obtain an overall estimate of relative strength (which then might be used to decide whether to keep escalating). Instead we assume that individuals only use the 0-1 outcome of each fight (whether they estimated themselves to be weaker) and integrate these outcomes of different fights directly in the willingness to escalate. In this way individuals can use information about their relative strength from different fights to decide whether to escalate without the necessity to obtain an overall estimate of their relative strength.

### Within-fight dynamics

Assumptions about within-fight dynamics are based on the sequential assessment game [26]. The main idea is that fights consist of multiple rounds in which each individual (1) incurs costs, which depend on its relative strength, (2) obtains noisy information about its relative strength, and (3) decides whether to give up, which happens when it is sufficiently certain to be weaker. The calculation of costs is motivated by the assumption that a weaker individual will incur greater costs. This might happen because a stronger individual can inflict more severe injuries to a weaker individual or because a weaker individual needs to put more effort in defending itself against a stronger opponent. We assumed that in each fighting round the costs *c*_*i *_for individual *i *are given by:

(6) $$c_{i}=e^{\left(s_{j}-s_{i}\right)}$$

where *s*_*i *_and *s*_*j *_are the strengths of individual *i *and its opponent *j*. Equation 6 ensures that the costs of the stronger individual are below one (but always stay above zero) and the costs of the weaker individual are above one (and equally matched opponents both incur costs of one).

Our implementation of the decision making process is based on the model of de Froment [32]. Thus, decision making is based on a single decision variable *d*, which is set to zero at the beginning of each fight and then updated based on estimated strength difference. In each fighting round *r *an individual *i *that fights against an opponent *j *obtains an estimation of its relative strength *Δs*_*i,j,r*_, which is given by

(7) $$\Delta s_{i,j,r}=s_{i}-s_{j}+\varepsilon$$

where *s*_*i *_and *s*_*j *_are the strengths of both individuals and *ε *corresponds to observation errors. In each fighting round these errors are independently drawn for each individual from a normal distribution with mean zero and standard deviation σ_*ε*_.

If the observation error is relatively large then an accurate estimation of relative strength can only be obtained by pooling the estimations of several rounds. This pooling is done by subtracting the estimated strength difference *Δs *in each round from the decision variable *d *(figure 4, note that this implementation differs from that of de Froment [32] who adds the estimated strength difference to the decision variable). Thus, the value of *d *can be interpreted as the amount of evidence that has been collected that an individual is weaker. Subtracting *Δs *from *d *has the effect that estimations of being stronger (positive values of *Δs*_*i,j,r*_) decrease the evidence of being weaker and estimations of being weaker (negative values of *Δs*_*i,j,r*_) increase the evidence of being weaker. Therefore, a positive value of *d*_*i *_provides evidence that an individual *i *is weaker and a negative value suggest that *i *is stronger. The larger the absolute value of *d *the more certain an individual can be to be the weaker or stronger, respectively. It is assumed that an individual *i *decides to give up fighting when its value of *d*_*i *_increases above its genetically determined giving up threshold *T*_*i *_(figure 4). This leads to the effect that lower values of *T*_*i *_lead to shorter fights because the weaker individual tends to give up earlier, but it also increases the risk that a stronger individual erroneously assumes itself to be weaker and gives up. In addition, fight length generally depends on strength difference of the opponents with large differences resulting on average in shorter fights and small differences resulting in longer fights (figure 4).

In each fighting round both individuals decide simultaneously whether to give up. This allows three possible outcomes: (1) neither gives up, which means the fights continues, (2) only one individual gives up and thus leaves the resource to the opponent, and (3) both give up, in which case one of them is randomly chosen as the winner who obtains the resource.

In each fighting round costs are subtracted from the fitness of individuals. In case the fitness of an individual drops below zero the individual is forced to give up in this round. In addition, individuals with negative fitness will set all their escalation probabilities *w*_*i,j *_to zero, which prevents them from initiating any escalated fights in the future.

## Appendix B: Additional Analyses

### Observation errors

To investigate how our results were affected by variation of the standard deviation σ_*ε *_of errors in estimating relative strength, we performed additional simulations in which the value of σ_*ε *_was set to 0.5 and 2. All other parameter values and performed analyses were identical to the description in section 2.2. *'Model Analyses'.*

**Figure 5:**
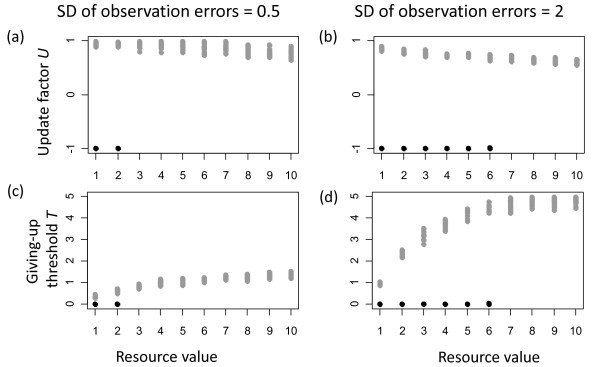
**Effects of changing the standard deviation σ_*ε *_of errors in estimating relative strength from 0.5 (a, c) to 2 (b, d) on evolved parameters**. Values that correspond to the evolved turn-taking strategy are shown in black and those corresponding to the contest strategy are shown in grey.

Changes of σ_*ε *_had a slight effect on evolved values of the update factor *U *(figure 5 a, b) and a strong effect on the evolved values of the giving-up threshold *T *(figure 5 c, d) for the contest strategy, but no apparent changes occurred for evolved parameters of the turn-taking strategy. In addition, increasing observation errors led to an increase in the range of benefits for which the turn-taking strategy evolved (figure 5).

Lower values of *T *and *U *of the contest strategy compensated higher observation errors and thus ensured the reliable identification of the stronger individual in each dyad. However, lower values of *T *led to longer and thus more costly fights and lower values of *U *led on average to more fights because individuals would more often fight not only in the first conflict but also in subsequent conflicts before 'agreeing' on a stable dominance-subordinate relationship.

The effectiveness of the contest strategy crucially depended on identifying which individual in a dyad was stronger. Larger observation errors made this process more costly and thus reduced the selection advantage of the contest strategy when it competed with the turn-taking strategy. In contrast, the turn-taking strategy did not depend on the identification of stronger individuals and thus was not affected by changes in observation errors. Taken together these effects explain why the turn-taking strategy evolved under a broader range of benefit values when observation errors were large.

### Number of conflicts

To investigate how our results were affected by variation in the number of conflicts, we performed additional simulations with number of conflict rounds set to 4 and 40. All other parameter values and performed analyses were identical to the description in section 2.2. *'Model Analyses'.*

**Figure 6:**
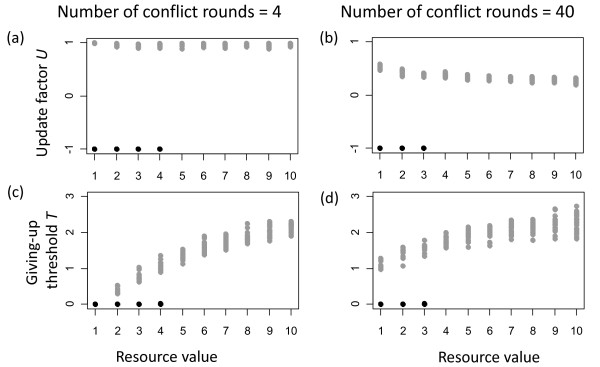
**Effects of changing the number of conflict rounds from 4 (a, c) to 40 (b, d) on evolved parameters**. Values that correspond to the evolved turn-taking strategy are shown in black and those corresponding to the contest strategy are shown in grey.

Changes in the number of conflict rounds had a strong effect on evolved values of the update factor *U *(figure 6 a, b) and a slight effect on the evolved values of the giving-up threshold *T *(figure 6 c, d) for the contest strategy, but no apparent changes occurred for evolved parameters of the turn-taking strategy. In addition, increasing the number of conflicts led to a decrease in the range of benefits for which the turn-taking strategy evolved (figure 6).

Individuals with the contest strategy escalated initial conflicts to costly fights to identify the stronger individual (who then became dominant). Thus, these initial conflicts were costly investments for the establishment of stable subordinate-dominance relationships. Investing a lot would ensure that the stronger individual is always identified (also when strength differences are small). However, larger investments would be also more costly. Evolutionary dynamics were finding optimized solutions for this trade off. How much individuals invested initially depended on how much they could gain later in their relationships. These gains increased with the number of conflict rounds. Therefore, larger investments were made when the number of conflict rounds was large, which is reflected by the lower values of *U *(which led to more frequent fights) and higher values of *T *(which led to longer fights).

Larger initial investments of the contest strategy for a larger number of conflict rounds increased the effectiveness of the contest strategy. In contrast, the turn-taking strategy remained unaffected by changes in the number of conflicts, which explains why this strategy evolved under a narrower range of benefit values when the number of conflict rounds was large.

### Group size

To investigate how our results were affected by variation of group size, we performed additional simulations with group sizes of 5 and 20. All other parameter values and performed analyses were identical to the description in section 2.2. *'Model Analyses'.*

**Figure 7:**
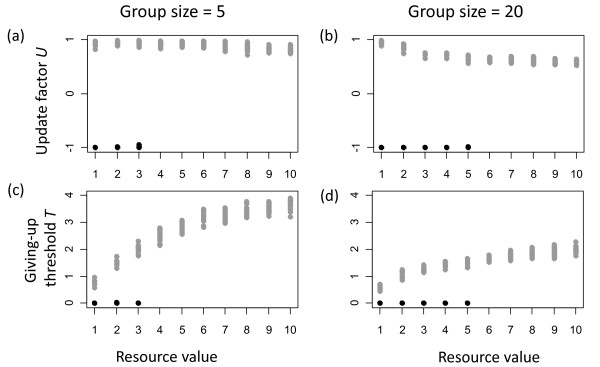
**Effects of changing group size from 5 (a, c) to 20 (b, d) on evolved parameters**. Values that correspond to the evolved turn-taking strategy are shown in black and those corresponding to the contest strategy are shown in grey.

Changes in group size affected the evolution of the update factor *U *(figure 7 a, b) and the giving-up threshold *T *(figure 7 c, d) for the contest strategy, but no apparent changes occurred for evolved parameters of the turn-taking strategy. In addition, increasing group size led to an increase in the range of benefits for which the turn-taking strategy evolved (figure 7).

At the first glance it might seem to be surprising that changes in group size led to changes in the evolution of the contest strategy given that all other parameters were held constant. The reason why these changes occurred in larger groups was an increased chance that two individuals of similar strength entered conflicts (because individual strength was drawn from a fixed distribution independent of group size). Smaller strength differences made it harder to identify the stronger individual in a dyad. Smaller differences generally led to longer and thus more costly fights (figure 4). This effect could be counterbalanced by a decrease in the giving-up threshold (which would lead to shorter fights) or an increase in the update factor (which would lead to less frequent fights). However, these changes would also result in a decreased likelihood to correctly identify the stronger individual in each dyad. Again evolutionary dynamics were optimizing this trade off in a way that with increasing group size the update factor and also the giving-up threshold decreased (figure 7), which led to shorter but more frequent fights.

Similar to the effects of increasing observation errors and decreasing the number of contests, larger groups decreased the effectiveness of the contest strategy, while the turn-taking strategy remained unaffected. This explains why an increasing group size allowed the turn-taking strategy to evolve under a broader range of benefit values.
